# Diagnostic and therapeutic challenge: A case of right ventricular thrombus

**DOI:** 10.21542/gcsp.2024.13

**Published:** 2024-03-03

**Authors:** Angad Bedi, Muhammad Sabri, Prerana Sevella, Hussam Al Hennawi, Gregory Pirolli

**Affiliations:** Department of Internal Medicine, Jefferson Abington Hospital, Abington, PA, USA

## Abstract

Right ventricular thrombus is a rare finding found in 4% of people diagnosed with pulmonary embolism. Although right ventricular thrombi are usually associated with atrial fibrillation, deep venous vein thrombi, or intracardiac procedures, isolated right ventricular thrombi are rare. Right ventricular thrombus has also been reported in patients with right ventricular infarction, as hypokinesis of the right ventricle causes blood stasis and promotes thrombosis as per Virchow’s triad. However, we present a case of isolated RV thrombus in a patient without evidence of deep vein thrombosis or right ventricular hypokinesis who presented with dyspnea on exertion.

## Case presentation

A 37-year-old female presented to the ED with symptoms of abdominal pain, lightheadedness, weakness, and dyspnea on exertion (DOE) for 1–2 days. The patient had a medical history of chronic iron deficiency anemia related to menorrhagia in the setting of uterine fibroids, adenomyosis, left-sided pneumonia (PNA)/empyema with left-sided video-assisted thoracoscopic surgery (VATS), and anxiety disorder.

On admission, she reported a menstrual cycle two days prior, and she had been bleeding through her pads every 2–3 h. She started to develop diffuse abdominal pain (waxing and waning in severity) since the onset of her period. She noticed increased abdominal distention and dysmenorrhea accompanied by nausea and vomiting for two days. Her last bowel movement occurred two days ago. She denied any blood in her stools or dysuria. The generalized weakness, fatigue, and DOE progressively worsened over the course of 2 days. She was taking ibuprofen and acetaminophen for pain; however, she experienced minimal relief. The patient denied any trauma or loss of consciousness (LOC). She reported having had similar abdominal pain and heavy periods since the age of 33 years, which had worsened over the past year.

**Figure 1.**
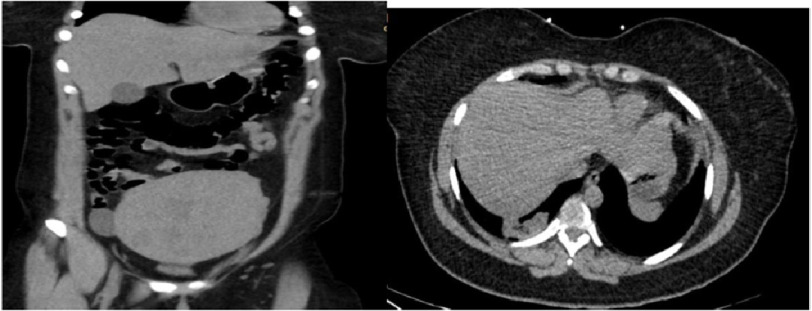
CTAP without contrast showing progressive uterine enlargement and heterogeneity. Stable 3.5 cm right adnexal/ ovarian cyst. New 5*2 cm mass like right lower lobe consolidation reflecting pneumonia.

**Figure 2.**
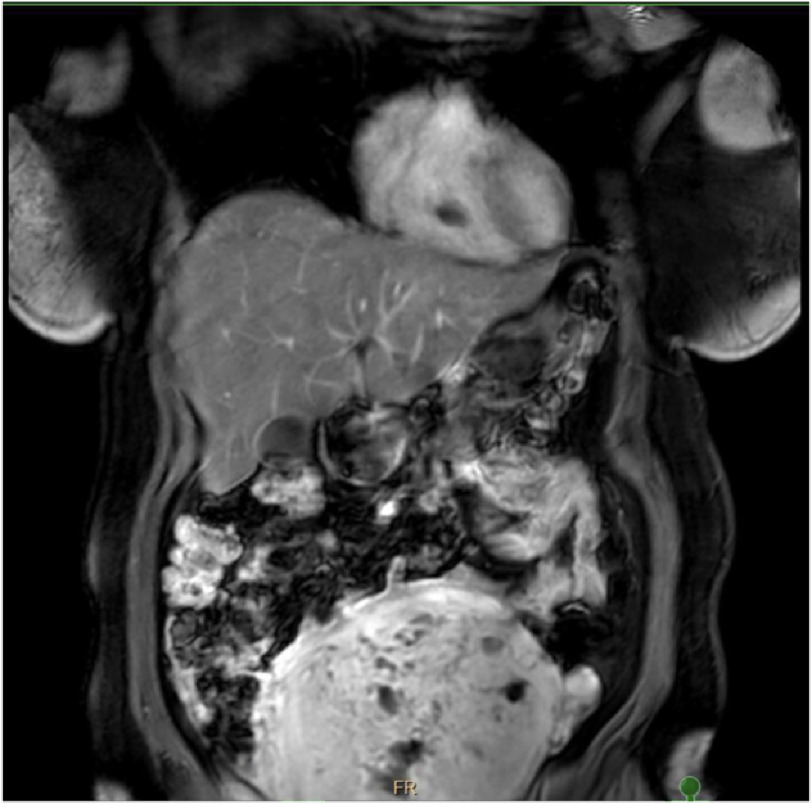
MRI AP with contrast shows an enlarged uterus with numerous cystic spaces consistent with diffuse uterine adenomyosis. Non-enhancing intra-cardiac mass in the right ventricle.

In the Emergency Department (ED), her blood pressure (BP) was measured as 135/87, heart rate (HR) 101 beats per minute (BPM), respiratory rate (RR) 18, temperature 99.6 F, and oxygen saturation 95% on room air. Physical examination revealed pale skin, tachycardia, decreased breath sounds at the lung bases (more decreased on the right side compared to the left side), distended abdomen with no rigidity, tenderness, rebound tenderness, normal bowel sounds, blood in the vaginal vault, and a 22-week enlarged uterus with palpable masses.

Initial investigations showed hemoglobin (Hgb) 5.9 g/dL, red blood cell (RBC) 2.75*10^∧^12, WBC 53600, MCV 68 fl, MCH 21.5 pg, MCHC 31.6 g/dL, RDW 18.6 percent, platelet 233000, iron 14 mcg/dL, ferritin 29 mcg/L, sodium (Na) 129 mg/dL, potassium (K) 4.2, chloride 91 mg/dL, anion gap 18, creatinine 3.07 mg/dL, eGFR 19 mL/min/1.73m^∧^2, glucose 215 mg/dL, and urinalysis positive for 5/Hpf RBC. Urine pregnancy test results were negative.

Computed tomography of the abdomen pelvis (CTAP) without contrast showed interval progressive uterine enlargement, a stable 3.5 cm right adnexal/ovarian simple cyst, and a 5 × 2 cm mass-like right lower lobe consolidation (Figure 1).

The patient was started on intravenous (IV) vancomycin and piperacillin/ tazobactam and was transfused with two units of packed RBCs. Obstetrics/gynecology was consulted for abnormal uterine bleeding, hematology for anemia and leukocytosis, pulmonology for right lower lobe consolidation, and nephrology for hyponatremia and AKI. Vancomycin and piperacillin/tazobactam were discontinued, and the patient was started on amoxicillin/clavulanate with azithromycin to treat the suspected community-acquired pneumonia. The anemia improved with daily intravenous iron administration. Fractional excretion of sodium was 0.9%, and AKI was managed with intravenous fluids.

Magnetic resonance imaging of the abdominal pelvis (MRI AP) with contrast shows an enlarged uterus with numerous cystic spaces consistent with diffuse uterine adenomyosis. However, it showed a non-enhancing mass in the right ventricle (Figure 2).

**Figure 3.**
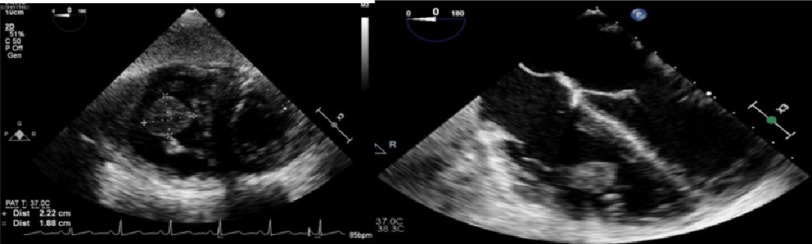
Transesophageal echocardiography shows a 2.2 * 1.9 cm mobile mass likely adherent to sub valvular chordae in the right ventricle.

Transthoracic echocardiography (TTE) showed normal right and left ventricular ejection fractions with no evidence of an intracardiac mass. However, transesophageal echocardiography (TEE) showed a 2.2 * 1.9 cm mobile mass in sub valvular chordae in the right ventricle with mild tricuspid regurgitation (TR) and mitral regurgitation (MR) (Figure 3).

## Differential diagnosis

The differential diagnosis for this mass includes right ventricular vegetation due to infective endocarditis and cardiac tumors, such as myxoma, fibroma, or intracardiac thrombus. The patient was hemodynamically stable and was managed for uterine bleeding, which halted the initiation of anticoagulation therapy.

## Investigation and management

Microbiological testing, including blood cultures, HIV, Coxiella burnetti, Brucella, Legionella pneumophila, fungal, and tuberculosis testing, were negative. Autoimmune profile tests for anti-nuclear antibody (ANA), anti-neutrophil cytoplasmic antibody (ANCA), anti-double-stranded DNA antibody, anti-cardiolipin antibody, lupus anticoagulant, anti-B2 glycoprotein, and rheumatoid factor (RF) were negative. Malignancy workup showed carcinoembryonic antigen (CEA) 1.1, elevated Ca 19-9 (294), and Ca- 125 (1510). Bilateral lower extremity Doppler (LE US) was insignificant for any thrombus, while D-dimer was 2703 and fibrinogen was 524.

**Figure 4.**
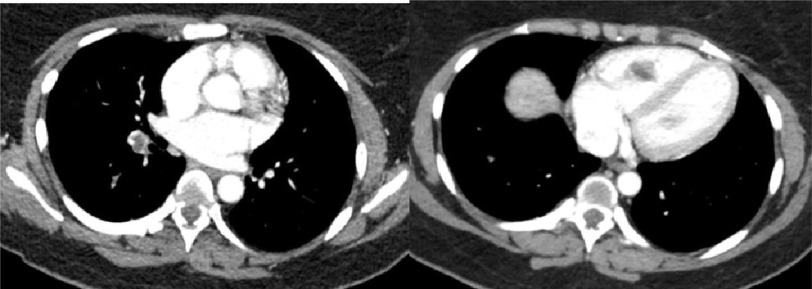
CT angiography shows right greater than left lower lobe pulmonary emboli with right heart strain and slightly increased right lower lobe consolidation from initial CT reflecting pulmonary infarct.

Cardiothoracic surgery (CTS) was consulted for possible operative management of this intracardiac mass. CT angiography (CTA) of the chest showed right greater than left lower lobe pulmonary emboli with right heart strain and right lower lobe consolidation from a right pulmonary infarct (Figure 4).

**Figure 5.**
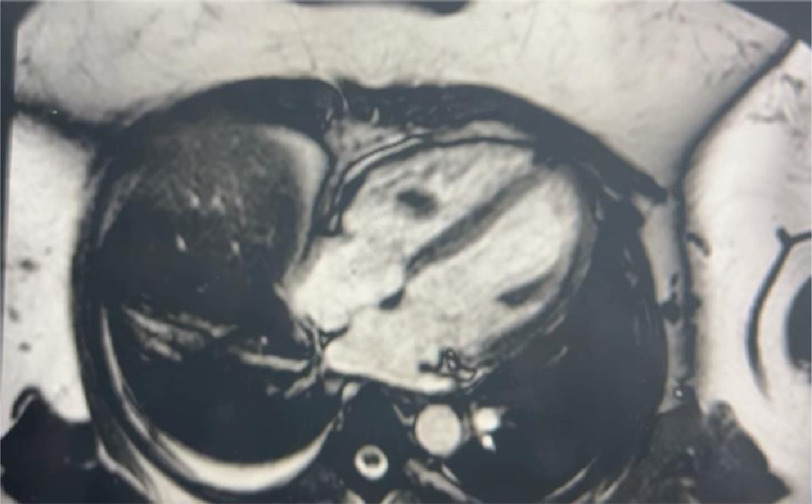
CMRI showing a mass in the right ventricle. There is no delayed myocardial enhancement to suggest scar, infarct, or infiltrative diseases.

**Figure 6.**
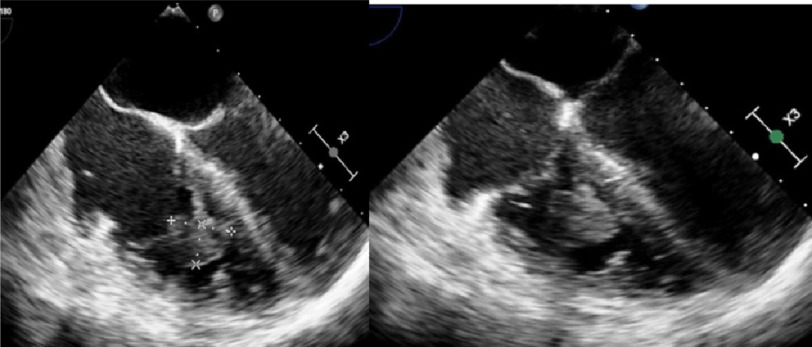
Repeat Transesophageal Echocardiography shows a large pedunculated, well-circumscribed mass in the tricuspid valve apparatus measuring from 2.1 cm to 1.8 cm. There was a small linear, mobile echo density visualized on the mass, which may represent a small thrombus.

CTS recommended cardiac MRI (cMRI), and the patient was started on heparin. CMRI revealed a mass in the right ventricle, consistent with a right ventricular thrombus (Figure 5).

Heparin was discontinued, and the patient was started on Eliquis. Repeat TEE showed a large pedunculated, well-circumscribed mass in the tricuspid valve apparatus measuring from 2.1 cm to 1.8 cm. There was a small linear, mobile echo density visualized on the mass, which may represent a small thrombus (Figure 6).

CTS recommended stopping Eliquis, and surgery was planned for right ventricle (RV) mass excision as there was no change in mass size despite anticoagulation. Open-heart surgery with excision of RV mass was performed. The pathology report revealed an organized fibrin, compatible with a thrombus. Anticoagulation therapy was started to decrease the risk of thromboembolism.

## Discussion

An intracardiac mass is more likely to be a thrombus, vegetation, or metastatic tumor and less likely to be a primary cardiac tumor^1^. Primary cardiac tumors are rare, and malignant tumors are much less common than benign ones. Most primary intracardiac tumors originate from the left atrium and rarely arise from the right ventricle. Myxomas are found in the left atrium in approximately 75% of cases and in the right atrium in 25% of cases. Angiosarcoma usually occurs in the right atrium^1^. Rhabdomyomas and fibromas are the most common primary cardiac tumors in young individuals.

Compared to left ventricular (LV) thrombus, RV thrombus is usually asymptomatic before complications such as PE or paradoxical stroke develop^2^. Although our patient had dyspnea on exertion, it improved after blood transfusion, indicating dyspnea secondary to anemia. The patient was incidentally found to have an RV thrombus and PE. Reported mortality from RHT ranges from 27% to 45% when treated, and almost 100% in untreated patients. Similar to our patient, almost 98% of RHTs are associated with PE^3^. Risk factors associated with right ventricular thrombus include previous bleeding events, younger age, cancer, congestive heart failure, transient systolic blood pressure <100 mmHg, episodes of syncope, and arterial oxyhaemoglobin saturation <90%^4^. Hypercoagulable states, such as Factor 5 Leiden and antithrombin 3 mutations, have been associated with RV thrombus^5^. However, hypercoagulable workup was negative in our patient. There are three types of RV thrombus: Type A thrombus or high-risk thrombus are thin, mobile, and frequently associated with PE. Type B thrombus is usually immobile and originates in situ. Type C thrombus is mobile but rare and has intermediate characteristics between type A and type B^6^. Our patient had type A thrombus but was asymptomatic and hemodynamically stable with no symptoms of right heart failure or hypoxemia.

Free-floating RHT (FFRHT) is rare, underdiagnosed in patients with PE, and represents a medical emergency^7^. The mortality rate in patients with RHT and PE is higher than that in those with PE alone^7^. FFRHT and PE were observed together. In a series by Chartier et al. ^8^, on the day of admission, almost 21.1% of patients died, and the hospital mortality rate was 44.7% in patients who had a large RHT due to the occurrence of a sudden pulmonary embolism.

Papillary muscle, moderator band, and coarse trabeculations in the RV make the diagnosis of right ventricular thrombus challenging^5^. TTE is the initial modality of choice for diagnosing RHT^8^. TEE can be performed when the diagnosis is doubt^8^. Although TTE and TEE are good modalities for diagnosing RV thrombus, other modalities, such as contrast-enhanced MRI (CMR), help delineate and characterize the lesion as a tumor, thrombus, or vegetation^6^. CMR offers accurate and non-examiner-dependent images. Considering T1-weighted (T1w), T1w with fat saturation, and T2-weighted (T2w) sequences, first-pass perfusion, early gadolinium enhancement(EGE), and late gadolinium enhancement (LGE), additional tissue characterization and detection of the vascularization of cardiac masses are possible. CMR also has high sensitivity and specificity for detecting intracardiac thrombus^6^. CMR revealed an intracardiac mass as a thrombus in our patient.

Treatment includes thrombolysis, anticoagulation, percutaneous retrieval technique, or surgical thrombectomy^7^. PE associated with Type A RV thrombus demands immediate thrombolysis or thrombectomy despite hemodynamic stability, while thrombolysis is not recommended for Type B thrombus^6^. Loherman et al. reported the case of RHT with bilateral proximal PE that was managed with thrombectomy^9^. This procedure can be performed in centers where thrombolysis is unavailable^7^. Puls et al. reported three cases of RHT. One patient was managed with surgical thrombectomy, while the remaining two were managed with thrombolysis ^10^.

It should also be noted that anticoagulation can sometimes not help in decreasing the size and resolving the right heart thrombus, as was observed in our patient. It is important to reimage the patient if the plan is to manage with only anticoagulation and not by surgical thrombectomy or thrombolysis, as in our patient. Managing patients with RHT alone with anticoagulation has more reported mortality than those managed with thrombolysis and surgical thrombectomy^7^. Surgical thrombectomy or thrombolysis can be followed by anticoagulation^7^. Athappan et al. reported an increase in mortality in patients managed with anticoagulation only compared to those managed with surgical thrombectomy or thrombolysis. Managing the elderly with only anticoagulation had satisfactory results, as they had an increased risk of mortality from bleeding when managed with thrombolysis.

## Conclusions

This case is unique in that an individual can have type A RHT and be asymptomatic, or presentation may be obscured when managing another acute condition. We present a case of RHT that was incidentally found during imaging for another condition. It is unique that our patient had a right ventricular thrombus without DVT, Afib, history of cardiomyopathy, or an intracardiac device. TTE is the first imaging modality of choice, although TEE and CMR are usually required to characterize the lesion further. RHT usually requires thrombolysis or surgical thrombectomy, and relying only on anticoagulation can defer the resolution and increase mortality risk. The decision to manage an intracardiac thrombus by only anticoagulation should also be followed by interval imaging and should be made after considering the risks of bleeding by thrombolysis and the benefits of resolution with only anticoagulation.
